# Exhaled carbon monoxide in asthmatics: a meta-analysis

**DOI:** 10.1186/1465-9921-11-50

**Published:** 2010-04-30

**Authors:** Jingying Zhang, Xin Yao, Rongbin Yu, Jianling Bai, Yun Sun, Mao Huang, Ian M Adcock, Peter J Barnes

**Affiliations:** 1 Department of Respiratory Disease, The First Affiliated Hospital of Nanjing Medical University, 300 Guangzhou Road, Nanjing, China; 2 Department of Epidemiology and Biostatistics, School of Public Health, Nanjing Medical University, 140 Hanzhong Road, Nanjing, China; 3 Airway Disease Section, National Heart and Lung Institute, Imperial College, Dovehouse Street, London, UK

## Abstract

**Background:**

The non-invasive assessment of airway inflammation is potentially advantageous in asthma management. Exhaled carbon monoxide (eCO) measurement is cheap and has been proposed to reflect airway inflammation and oxidative stress but current data are conflicting. The purpose of this meta-analysis is to determine whether eCO is elevated in asthmatics, is regulated by steroid treatment and reflects disease severity and control.

**Methods:**

A systematic search for English language articles published between 1997 and 2009 was performed using Medline, Embase and Cochrane databases. Observational studies comparing eCO in non-smoking asthmatics and healthy subjects or asthmatics before and after steroid treatment were included. Data were independently extracted by two investigators and analyzed to generate weighted mean differences using either a fixed or random effects meta-analysis depending upon the degree of heterogeneity.

**Results:**

18 studies were included in the meta-analysis. The eCO level was significantly higher in asthmatics as compared to healthy subjects and in intermittent asthma as compared to persistent asthma. However, eCO could not distinguish between steroid-treated asthmatics and steroid-free patients nor separate controlled and partly-controlled asthma from uncontrolled asthma in cross-sectional studies. In contrast, eCO was significantly reduced following a course of corticosteroid treatment.

**Conclusions:**

eCO is elevated in asthmatics but levels only partially reflect disease severity and control. eCO might be a potentially useful non-invasive biomarker of airway inflammation and oxidative stress in nonsmoking asthmatics.

## Background

Asthma is a chronic inflammatory disorder of the airways, associated with airway hyperresponsiveness (AHR) that leads to recurrent episodes of wheezing, breathlessness, chest tightness, and coughing, particular at night or in the early morning [1]. The assessment of airway inflammation is likely to play an important role in the future management of asthma. However, the "gold standard" for assessment of airway inflammation, bronchial biopsy, is invasive and too expensive to perform on a regular basis. Hence, much attention is paid to the non-invasive measurement of airway inflammation, such as induced sputum and of surrogates such as exhaled nitric oxide (eNO).

The level of eNO is elevated in asthmatics, even when asymptomatic [2-4], and shows a good response to corticosteroids [5,6]. However, the measurement of eNO is expensive, limiting its use in primary care and most specialist clinics.

Some studies have reported that exhaled carbon monoxide (eCO), which is used as an indicator of smoking [7], is significantly increased in asthmatics not treated with corticosteroids [8,9]. CO in the body is mainly derived from the degradation of hemoglobin by the enzyme heme oxygenase (HO) [10]. It has been found that the expression of the inducible isoform, HO-1, is increased in alveolar macrophages in asthmatics in association with increased exhaled CO [9]. These findings suggested that eCO may be a candidate of non-invasive biomarker of airway inflammation.

However, several studies have failed to replicate these early results [11,12]. We therefore performed a meta-analysis of the published evidence to determine whether eCO is increased in asthmatic adults and children, and whether eCO may reflect the clinical severity or be influenced by corticosteroids.

## Methods

A comprehensive literature search from 1997 to 2009 in English language was performed in Medline (using PubMed as the search engine), Embase and Cochrane databases. The search terms were 'exhaled CO', 'expired CO', 'carbon monoxide', 'asthma', 'bronchial spasm', 'bronchoconstriction', 'bronchial hyperreactivity', 'airway inflammation', 'wheeze' and 'wheezing'. (Additional file 1)

A priori inclusion criteria was: 1) observational studies; 2) comparing eCO levels between adults or children with an established diagnosis of asthma and healthy control subjects or comparing eCO levels in the same asthmatic population before and after steroid treatment; 3) both asthmatic patients and healthy subjects were nonsmokers, since smoking status could significantly affect eCO levels [7]. Exclusion criteria included: 1) asthmatic patients with systemic diseases, such as diabetes, sepsis [13,14] and 2) studies without healthy subjects as the control group.

A systematic search including a title screen, abstract review, and full text article review. Each study was evaluated independently by two investigators (JZ and YS). Any disagreements about adjudications were resolved through the third party (XY). For each eligible study, we extracted data including number of participants (n), age, gender, clinical feature, treatment, method measuring eCO, and the mean value (mean), standard deviation (SD), 95% confidence interval (CI), or standard error of mean (SEM) of exhaled CO. SEM or 95%CI was transformed to SD, using standard statistical conversions.

Review Manager (Version 5, The Cochrane Collaboration) was used to generate the weighted mean differences (WMD) of eCO between asthmatics and healthy subjects. *I*^2 ^statistic was tested for heterogeneity. We used a fixed effects model when *I*^2 ^was equal or less than 50%, and a random effects model when *I*^2 ^was greater than 50%. Meta-regression analysis on the time of publication was performed by STATA (Version 9.2, STATA Corporation, College Station). Subgroup analysis was performed, according to: 1) whether the study group consisted of adult or children, 2) whether the participants were treated with corticosteroids (systemic or inhaled), 3) the clinical severity of asthma. Publication bias was evaluated through visual inspection of funnel plots, the Begg's test [15] and the Egger's Asymmetry test [16], calculating by STATA. Publication bias may be present if P value is less than 0.05.

## Results

**Figure 1:**
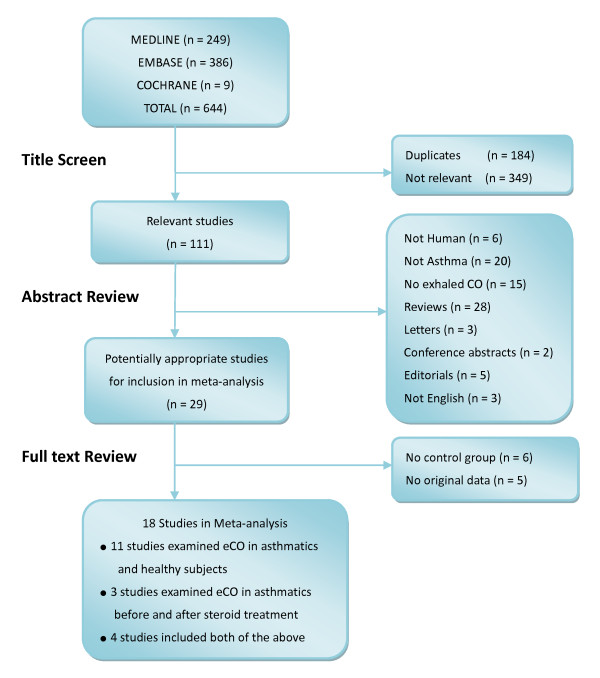
**Results of the systematic literature search**.

**Figure 2:**
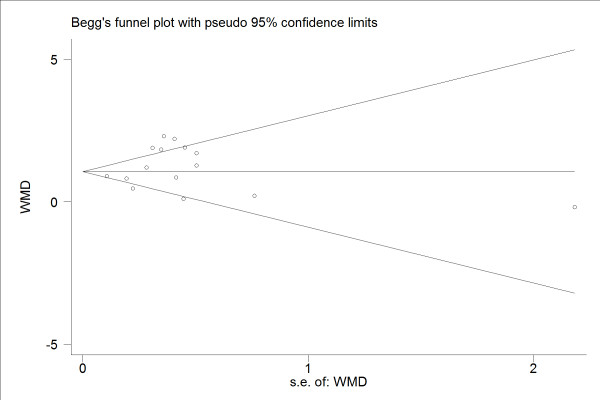
**Begg's funnel Plot**.

Figure 1 shows the results of the search for articles. Of the total 644 articles, 184 duplicates and 349 irrelevant studies were excluded after screening the titles. A further 82 articles failed to meet the inclusion criteria after abstract review. Among the remaining 29 articles, 6 studies did not have healthy control groups and 5 studies did not provide sufficient eCO data such as mean value, SD, 95%CI or SEM. We subsequently tried to contact the authors by email to obtain further information without success. Finally, 18 articles were included in our meta-analysis [17-34]. 4 studies directly provided SD of eCO, 2 studies used 95%CI and the remaining studies provided SEM. As no unequal variances were mentioned in these studies, the classical t-test was used to transform 95%CI into SD. The characteristics of the 18 studies were listed in the Additional file 2, 3. There were no evidence of publication bias (Begg's test: P = 0.843; Egger's Asymmetry test: P = 0.211; Figure 2 shows the Funnel plot). The heterogeneity between different studies is significant (*I*^2 ^= 75%), therefore, we performed a meta-regression analysis on the time of publication. The τ^2 ^estimate is 0.3782, which means the time of publication could only partly explain the heterogeneity.

### 1. Overall eCO levels in asthmatics and healthy subjects

**Figure 3:**
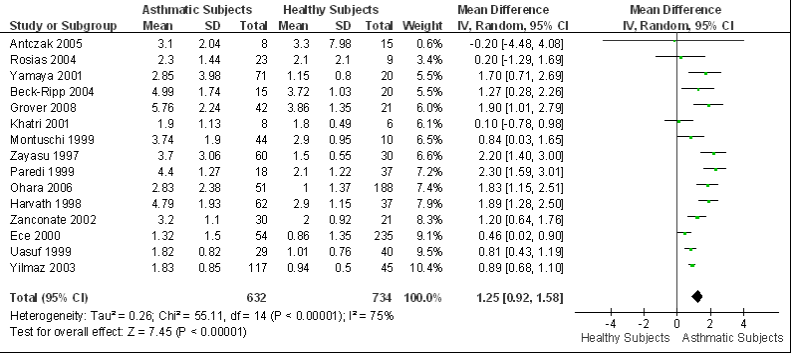
**Overall eCO levels in asthmatics**. The WMD of eCO levels between asthmatics and healthy subjects is 1.25 ppm (95%CI: 0.92 to 1.58) using the random effects model. Test for heterogeneity *I*^2 ^= 75%. SD: standard deviation; IV: inverse variance; CI: confidence interval; WMD: weighted mean difference; eCO: exhaled carbon monoxide.

Data was pooled from 15 studies irrespective of the age of the participants, the severity of disease or treatment. The overall WMD of eCO between asthmatics and healthy subjects was 1.25 ppm (95% CI 0.92 to 1.58; *I*^2 ^= 75%; Random effects model). Asthmatic patients showed significantly higher eCO levels as compared to healthy subjects. (Figure 3)

### 2. eCO levels in childhood and adult asthma

**Figure 4:**
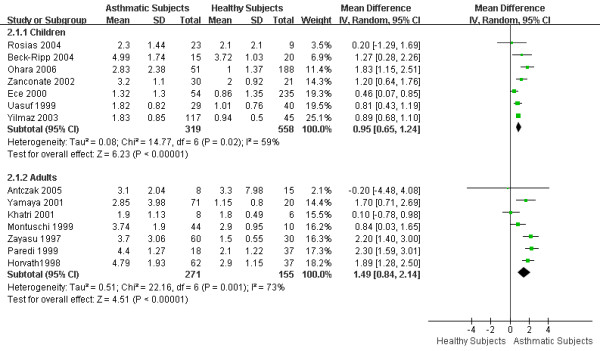
**eCO levels in childhood and adult asthma**. The WMD of eCO levels between asthmatic children and healthy children is 0.95 ppm (95%CI: 0.65 to 1.24) using the random effects model. Test for heterogeneity *I*^2 ^= 59%. The WMD of eCO levels between asthmatic adults and healthy adults is 1.49 ppm (95%CI: 0.84 to 2.14) using random effects model. Test for heterogeneity *I*^2 ^= 73%. SD: standard deviation; IV: inverse variance; CI: confidence interval; WMD: weighted mean difference; eCO: exhaled carbon monoxide.

There were 7 articles focused on childhood asthma and 7 on adult asthma. The remaining article [31] studied a population above 10 years old and, therefore, could not be included as either childhood or adult asthma. The WMD of eCO between asthmatic children and healthy children was 0.95 ppm (95% CI 0.65 to 1.24; *I*^2 ^= 59%; Random effects model). And the WMD of eCO between asthmatic adults and healthy adults was 1.49 ppm (95% CI 0.84 to 2.14; *I*^2 ^= 73%; Random effects model). Both asthmatic children and asthmatic adults showed significant higher eCO levels compared to healthy children and healthy adults respectively. (Figure 4)

### 3. eCO levels in steroid-free and steroid-treated asthmatics

**Figure 5:**
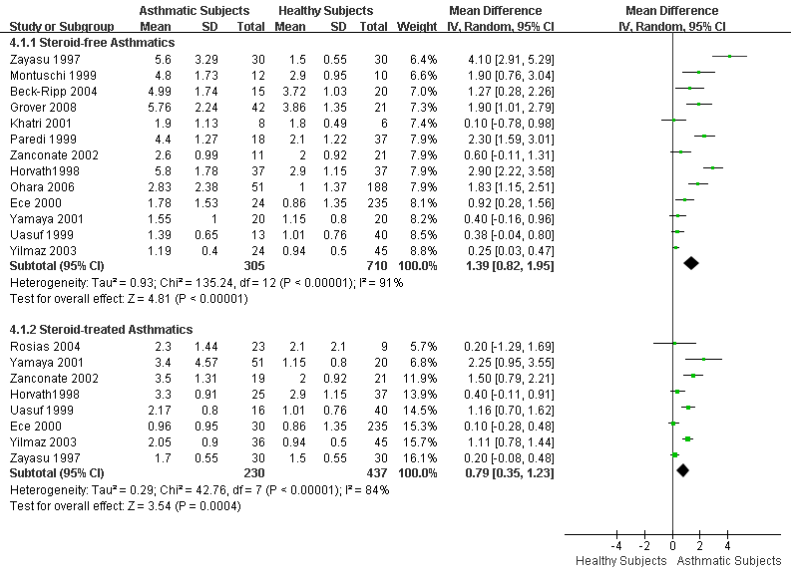
**eCO levels in steroid-free and steroid-treated asthmatics**. The WMD of eCO levels between steroid-free asthmatics and healthy subjects is 1.39 ppm (95%CI: 0.82 to 1.95) using the random effects model. Test for heterogeneity *I*^2 ^= 91%. The WMD of eCO levels between steroid-treated asthmatics and healthy adults is 0.79 ppm (95%CI: 0.35 to 1.23) using the random effects model. Test for heterogeneity *I*^2 ^= 84%. SD: standard deviation; IV: inverse variance; CI: confidence interval; WMD: weighted mean difference; eCO: exhaled carbon monoxide.

13 articles mentioned steroid-free asthmatics (defined as either steroid naïve or not receiving regular inhaled or systemic corticosteroids and treated by on-demand β_2_-agonist), and 8 articles mentioned steroid-treated asthmatics (defined as current use of inhaled or systemic corticosteroids irrespective of β_2_-agonist or montelukast use). The WMD of eCO was 1.39 ppm (95%CI 0.82 to 1.95; *I*^2 ^= 91%; Random effects model) between steroid-free asthmatics and healthy subjects, and 0.79 ppm (95%CI 0.35 to 1.23; *I*^2 ^= 84%; Random effects model) between steroid-treated asthmatics and healthy subjects. Both steroid-free and steroid-treated asthmatics had significantly higher eCO levels than healthy subjects. Steroid-treated asthmatic patients had lower eCO levels compared to steroid-free asthmatic patients (0.79 ppm vs 1.39 ppm) but this failed to reach statistical significance. (Figure 5)

### 4. eCO levels in different asthma severities

**Figure 6:**
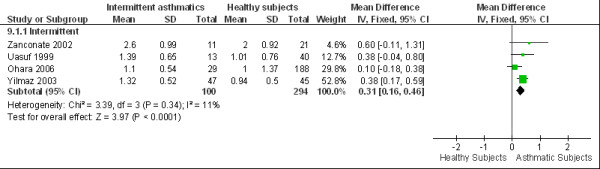
**eCO levels in intermittent asthmatics**. The WMD of eCO levels between intermittent asthmatics and healthy subjects is 0.31 ppm (95%CI: 0.16 to 0.46) using the fixed effects model. Test for heterogeneity *I*^2 ^= 11%. SD: standard deviation; IV: inverse variance; CI confidence interval; WMD: weighted mean difference; eCO: exhaled carbon monoxide.

**Figure 7:**
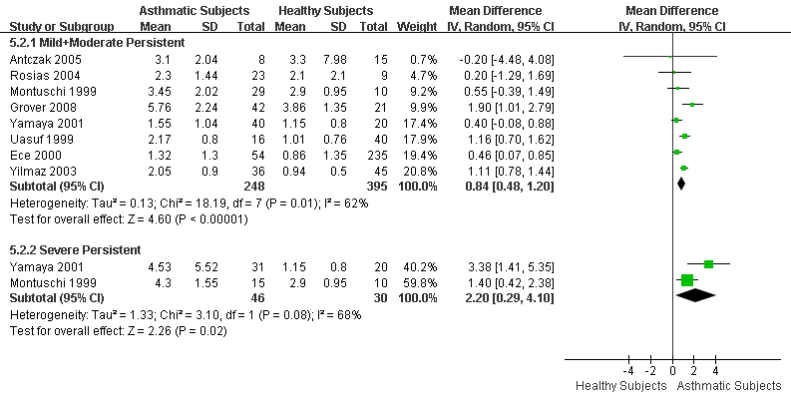
**eCO levels in mild + moderate persistent and severe persistent asthmatics**. The WMD of eCO levels between mild + moderate persistent asthmatics and healthy subjects is 0.84 ppm (95%CI: 0.48 to 1.20) using the random effects model. Test for heterogeneity *I*^2 ^= 62%. The WMD of eCO levels between severe persistent asthmatics and healthy subjects is 2.20 ppm (95%CI: 0.29 to 4.10) using the random effects model. Test for heterogeneity *I*^2 ^= 68%. SD: standard deviation; IV: inverse variance; CI: confidence interval; WMD: weighted mean difference; eCO: exhaled carbon monoxide.

According to the GINA [1] criteria, there are 4 categories of disease severity: intermittent, mild persistent, moderate persistent and severe persistent asthma. However, due to the limited information provided by the articles, we could not differentiate mild persistent asthma from moderate persistent asthma. Therefore, we separated the subjects into three groups: intermittent asthma (4 articles), mild + moderate persistent asthma (8 articles) and severe persistent asthma (2 articles). The WMD of eCO was 0.31 ppm (95%CI 0.16 to 0.46; *I*^2 ^= 11%; Fixed effects model) between intermittent asthmatics and healthy subjects (Figure 6), 0.84 ppm (95%CI 0.48 to 1.20; *I*^2 ^= 62%; Random effects model) between mild + moderate persistent asthmatics and healthy subjects, and 2.20 ppm (95% CI 0.29 to 4.10; *I*^2 ^= 68%; Random effects model) between severe persistent asthmatics and healthy subjects (Figure 7). The eCO levels were higher across all asthma severities as compared to healthy subjects. Furthermore, both mild + moderate persistent and severe persistent asthmatics showed significantly higher eCO levels than intermittent asthmatics. However, the eCO level of mild + moderate persistent asthma did not differ significantly from that of severe persistent asthma.

### 5. eCO levels in different asthma control states

**Figure 8:**
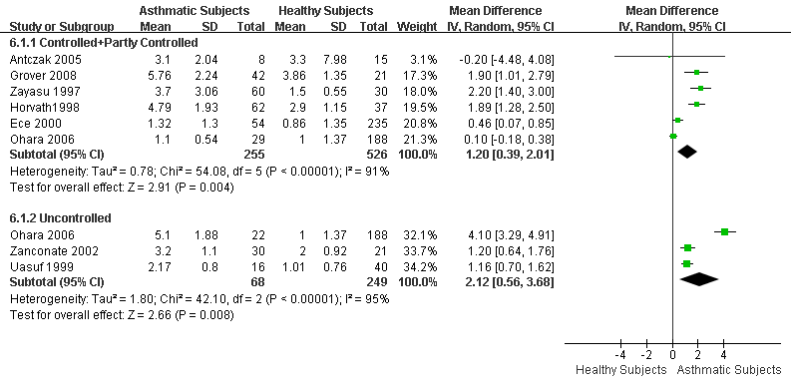
**eCO levels in different asthma control states**. The WMD of eCO levels between controlled + partly controlled asthmatics and healthy subjects is 1.20 ppm (95%CI: 0.39 to 2.01) using the random effects model. Test for heterogeneity *I*^2 ^= 91%. The WMD of eCO levels between uncontrolled asthmatics and healthy adults is 2.12 ppm (95%CI: 0.56 to 3.68) using the random effects model. Test for heterogeneity I^2 ^= 95%. SD: standard deviation; IV: inverse variance; CI: confidence interval; WMD: weighted mean difference; eCO: exhaled carbon monoxide.

We attempted to classify asthma control status into 3 categories: controlled, partly controlled and uncontrolled [1]. However, some studies did not provide enough information to distinguish between controlled and partly controlled disease. Thus, we divided subjects into 2 groups: controlled + partly controlled group (6 articles) and uncontrolled group (3 articles). The WMD of eCO was 1.20 ppm (95%CI 0.39 to 2.01; *I*^2 ^= 91%; Random effects model) between controlled + partly controlled asthmatics and healthy subjects, and 2.12 ppm (95%CI 0.56 to 3.68; *I*^2 ^= 95%; Random effects model) between uncontrolled asthmatics and healthy subjects. Both controlled + partly controlled asthma and uncontrolled asthma showed significantly higher eCO levels as compared to healthy subjects. There was no statistical difference in eCO levels between controlled + partly controlled and uncontrolled asthma. (Figure 8)

### 6. eCO levels in asthmatics before and after steroid treatment

**Figure 9:**
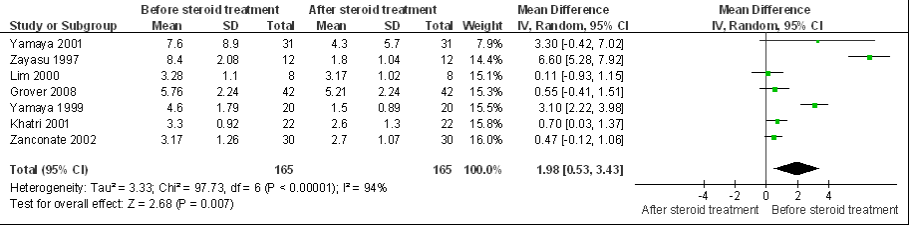
**eCO levels before and after steroid treatment**. The WMD of eCO levels before and after steroid treatment is 1.98 ppm (95%CI: 0.53 to 3.43) using the random effects model. Test for heterogeneity *I*^2 ^= 94%. SD: standard deviation; IV: inverse variance; CI: confidence interval; WMD: weighted mean difference; eCO: exhaled carbon monoxide.

There were 7 articles focusing on the influence of corticosteroid treatment on eCO, including 4 studies with inhaled corticosteroids, 2 studies with oral glucocorticoids and the remaining study with both inhaled and oral corticosteroids (the details of the studies are shown in Additional file 3). The WMD of eCO in asthmatics before and after steroid treatment was 1.98 ppm (95%CI 0.53 to 3.43; *I*^2 ^= 94%; Random effects model) indicating that eCO was significantly reduced by steroid treatment. (Figure 9)

## Discussion

**Figure 10:**
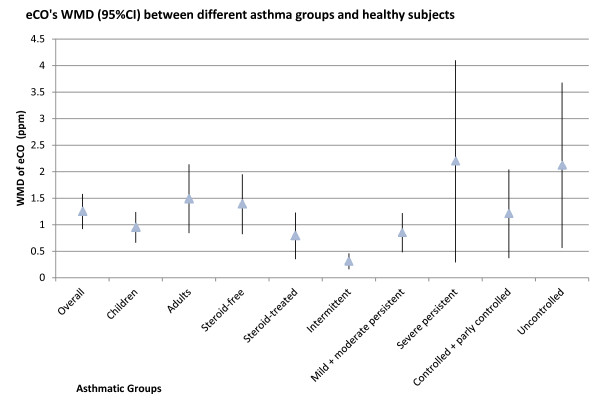
**eCO WMD (95%CI) between different asthma groups and healthy subjects**. There were no significant differences in eCO levels between steroid-free and steroid-treated asthmatics, mild + moderate persistent and severe persistent asthma, controlled + partly controlled and uncontrolled asthma. However, the eCO level of intermittent asthma was significantly lower than mild + moderate persistent asthma. CI: confidence interval; WMD: weighted mean difference; eCO: exhaled carbon monoxide; ▲: mean of WMD between asthmatics and healthy subjects; vertical line: 95%CI of WMD between asthmatics and subjects.

This meta-analysis based on 15 studies in 632 asthmatics and 734 healthy subjects demonstrates that eCO was increased in both adults and children with asthma, irrespective of steroid treatment, disease severity or level of asthma control (Figure 10). Apart from asthma, other diseases such as allergic rhinitis, bronchiectasis, lower and upper respiratory tract infections, interstitial lung disease and cystic fibrosis have also shown an increased production of eCO [10]. eCO was even found to be elevated in asymptomatic atopic subjects [35]. These findings suggest that increased eCO level may be an indicator of airway inflammation but it does not discriminate between diseases.

Endogenous CO in the expired air is mainly derived from the degradation of hemoglobin by HO [10]. Many mediators present in inflamed airways, including interleukin-1β, tumor necrosis factor-α, interferon-γ and hydrogen peroxide could induce HO-1 [35]. In vivo challenge with allergen, but not methacholine, also results in increased eCO levels, suggesting that an inflammatory response and not bronchoconstriction *per se *is necessary to enhance HO-1 activity and/or expression [36]. HO-1 is present in alveolar macrophages; however, there is conflicting evidence as to whether HO-1 is expressed in the airway epithelium and whether HO-1 is up-regulated in asthmatics [9,37,38]. Harju *et al *[38] showed that HO-1 was up-regulated in the alveolar macrophages of steroid-free asthmatics, and Horvath *et al *[9] also found increased expression of HO-1 in alveolar macrophages in asthmatics associated with increased exhaled CO. In contrast, Lim *et al *[37] demonstrated that HO-1 was equally expressed in alveolar macrophages from asthmatics and healthy subjects. This discrepancy was explained by the possibility that the airway was already exposed to daily oxidant stresses such as environment pollutants, and therefore the expression of HO-1 was probably already maximal in asthmatics and healthy subjects. Alternatively, the techniques used may not have been sensitive enough to quantify differences when HO-1 was highly expressed and enzymic activity was not measured.

The level of eCO in intermittent asthmatics was significantly lower than mild + moderate persistent and severe persistent asthmatics, suggesting that eCO may be correlated with disease severity. However, no statistical significance was found between mild + moderate persistent asthmatics and severe persistent asthmatics. There was much greater variability in the 95%CI of WMD for eCO in severe persistent asthma compared with other groups. This probably reflects the fact that only 2 studies were included in this group and the sample size (46 severe persistent asthmatics and 30 healthy subjects) was small, which decreased the statistical power. A similar reason probably underlies the variability in the uncontrolled asthma group. Thus, further investigation is needed to address whether eCO could reflect asthma control status.

Our meta-analysis demonstrated that the eCO level in steroid-treated asthmatics was not significantly different from that in steroid-free subjects although the average level of eCO is increased in steroid-free subjects. The lack of statistical significance may relate to the small sample size. In addition, this may possibly also reflect the wide variability in airway inflammation seen in subjects with differing degrees of asthma severity and degrees of control, or the fact that steroids may not affect HO-1 activity or expression. However, our meta-analysis did demonstrate a significant effect of a formal steroid intervention on eCO levels supporting in vitro data showing that steroids are able to directly down-regulate HO-1 promoter activity and expression [39].

Although steroid treatment could reduce the eCO concentrations in asthmatics, the eCO response might be less sensitive to steroid treatment than that of eNO [26,33]. Recent studies demonstrated that using eNO to guide treatment did not result in lower exacerbation frequency as compared to traditional asthma management [40,41]. It is possible that the eNO level is too sensitive to steroid treatment, and therefore, the underlying inflammation may still persist despite a significantly decreased eNO level. Hence, treatment based on the less steroid-sensitive eCO level, which correlates with asthma severity to some degree and is reduced after steroid treatment, could be of benefit.

There are some further limitations to using eCO as an indicator of airway inflammation. The most obvious one is that active and passive smoking status can remarkably affect the eCO level. Both healthy subjects and asthmatic subjects who were current smokers showed significantly higher eCO levels than their nonsmoking control subjects [42,43]. The eCO level of some healthy smoking subjects could be much higher than the level of nonsmoking severe persistent asthmatics reported previously [25]. Furthermore, Ece *et al *[23], demonstrated a significant relationship between passive smoking and eCO concentration in both healthy and asthmatic children. Thus, when using eCO levels to assess airway inflammation, the active and passive smoking status must be taken into consideration.

Another major limitation is the lack of standardization of eCO measurement. One study included in the meta-analysis used an infrared detection method and the others used an electrochemical method. There was no significant difference in WMD between asthmatics and healthy subjects when the study using infrared method was included (WMD: 1.25 ppm; 95%CI: 0.92 to 1.58; Random effects model; *I*^2 ^= 75%) or not (WMD: 1.33 ppm; 95%CI: 0.99 to 1.66; Random effects model; *I*^2 ^= 74%). Since the eCO values of nonsmoking healthy subjects in this meta-analysis are similar to those reported previously [42] we did not exclude studies due to their different measurements.

## Conclusions

eCO is elevated in non-smoking asthmatics and correlates with disease severity to some extent. Whether eCO reflects asthma control requires further investigation. Although eCO could not distinguish steroid-treated from steroid-free patients in a cross-sectional analysis, formal steroid treatment significantly reduced eCO levels. eCO might be a potentially useful non-invasive biomarker of airway inflammation and oxidative stress in nonsmoking asthmatics but further work is needed in this area.

## List of Abbreviations used

eCO: exhaled carbon monoxide; AHR: airway hyperresponsiveness; eNO: exhaled nitric oxide; HO: heme oxygenase; ppm: parts per million; SD: standard deviation; CI: confidence interval; SEM: standard error of mean; WMD: weighted mean difference; IV: inverse variance.

## Competing interests

The authors declare that they have no competing interests.

## Authors' contributions

All authors read and met ICMJE criteria for authorship. JZ, MH, XY and RY designed this study. JZ, XY and YS extracted data. JZ, XY, RY, JB performed the analysis. JZ wrote the first draft of the manuscript. XY, IMA and PJB critically revised. All authors read and approved the final manuscript.

## Supplementary Material

Additional file 1 **Search Strategy**. Document detailing the search strategy used. Click here for file

Additional file 2 **Studies included in the meta-analysis examining eCO levels in asthmatics and healthy subjects**. Data are expressed as #mean ± SEM; *mean ± SD; +95%CI; M: male, F: female, n: the number of participants, ppm: parts per million, FEV_1_: forced expiratory volume in one second; eCO: exhaled carbon monoxide; ICS: inhaled corticosteroids; L: low dose of inhaled corticosteroids; M: medium dose of inhaled corticosteroids; H: high dose of inhaled corticosteroids. Click here for file

Additional file 3 **Studies included in the meta-analysis examining eCO levels in asthmatics before and after steroid treatment**. Data are expressed as #mean ± SEM; *mean ± SD; +95%CI; M: male, F: female, n: the number of participants, ppm: parts per million, FEV_1_: forced expiratory volume in one second; eCO: exhaled carbon monoxide; ICS: inhaled corticosteroids; L: low dose of inhaled corticosteroids; M: medium dose of inhaled corticosteroids; H: high dose of inhaled corticosteroids. Click here for file
