# Supplementary material for: Exhaled carbon monoxide in asthmatics: a meta-analysis
Source: Respir Res. 2010 Apr 30;11(1):50. doi: 10.1186/1465-9921-11-50 (PMC2874770; doi:10.1186/1465-9921-11-50)
Supplement: Additional file 1 — Search Strategy. Document detailing the search strategy used. [file 1465-9921-11-50-S1.DOC]

1. Medline (using Pubmed as the search engine)

| #1 “exhaled CO”[tiab]  #2 “expired CO”[tiab]  #3 “carbon monoxide”[tiab] or “carbon monoxide”[mh]  #4 #1 OR #2 OR #3  #5 “asthma”[tiab] or asthma[mh]  #6 “bronchial spasm”[tiab] or “bronchial spasm”[mh]  #7 “bronchoconstriction”[tiab] or bronchoconstriction[mh]  #8 “bronchial hyperreactivity”[tiab] or “bronchial hyperreactivity”[mh]  #9 “airway inflammation”[tiab]  #10 wheeze[tiab] or wheezing[tiab]  #11 #5 OR #6 OR #7 OR #8 OR #9 OR #10  #12 #4 AND #11 Limits: Publication Data from 1997 to 2009 |
| --- |

1. Embase

| #1 ‘exhaled CO’:ab,ti  #2 ‘expired CO’:ab,ti  #3 ‘carbon monoxide’/exp  #4 #1 OR #2 OR #3  #5 ‘asthma’/exp  #6 ‘bronchial spasm’/exp  #7 ‘bronchoconstriction’/exp  #8 ‘bronchial hyperreactivity’/exp  #9 ‘wheezing’/exp  #10 ‘airway inflammation’:ab,ti  #11 #5 OR #6 OR #7 OR #8 OR #9 OR #10  #12 #4 AND #11 AND [1997-2009]/py |
| --- |

1. Cochrane

| #1 ‘exhaled CO’: ti, ab, kw  #2 ‘expired CO’:ti,ab,kw  #3 MeSH descriptor Carbon Monoxide explode all trees  #4 #1 OR #2 OR #3  #5 MeSH descriptor Asthma explode all trees  #6 MeSH descriptor Bronchial Spasm explode all trees  #7 MeSH descriptor Bronchoconstriction explode all trees  #8 MeSH descriptor Bronchial hyperreactivity explode all trees  #9 ‘airway inflammation’: ti, ab, kw  #10 ‘wheeze’: ti,ab, kw OR ‘wheezing’: ti, ab, kw  #11 #5 OR #6 OR #7 OR #8 OR #9 OR #10  #12 #4 AND #11, from 1997 to 2009 |
| --- |
