# Supplementary material for: Exhaled carbon monoxide in asthmatics: a meta-analysis
Source: Respir Res. 2010 Apr 30;11(1):50. doi: 10.1186/1465-9921-11-50 (PMC2874770; doi:10.1186/1465-9921-11-50)
Supplement: Additional file 2 — Studies included in the meta-analysis examining eCO levels in asthmatics and healthy subjects. Data are expressed as #mean ± SEM; *mean ± SD; +95%CI; M: male, F: female, n: the number of participants, ppm: parts per million, FEV1: forced expiratory volume in one second; eCO: exhaled carbon monoxide; ICS: inhaled corticosteroids; L: low dose of inhaled corticosteroids; M: medium dose of inhaled corticosteroids; H: high dose of inhaled corticosteroids. [file 1465-9921-11-50-S2.DOC]

| **Study** | **n** | **M/F** | **Age** | **Group** | **Clinical Feature** | **FEV_1_%predicted** | **Treatment** | **Measurement** | **eCO(ppm)** |
| --- | --- | --- | --- | --- | --- | --- | --- | --- | --- |
| Zayasu  1997^#^  Japan  [18] | 30 | 15/15 | 41±3 | Steroid-free | Airway obstruction was stable for at least 2 weeks before the study | 92±5 | β_2_-agonist | EC_50_ Smokerlyzer  Breath hold for 20s | 5.6±0.6 |
|  | 30 | 17/13 | 41±3 | Steroid-treated |  | 98±5 | ICS(L.M.H) |  | 1.7±0.1 |
|  | 30 | 11/19 | 43±3 | Healthy | ---- | --- | --- |  | 1.5±0.1 |
| Horvath  1998^+^  UK  [19] | 37 | 20/17 | 26.7-37.3 | Steroid-free | Stable for at least 2 weeks before the study | 87.9-100.1 | no/β_2_-agonist | EC_50_ MICRO Smokerlyzer | 5.20-6.39 |
|  | 25 | 11/14 | 30.3-41.5 | Steroid-treated |  | 70.0-83.9 | ICS(L.M.H) |  | 2.92-3.67 |
|  | 37 | 20/17 | 27.5-38.6 | Healthy | --- | --- | --- |  | 2.51-3.28 |
| Montuschi  1999^#^  UK  [20] | 12 | 7/5 | 27.8±1.34 | Mild persistent | ---- | 90±3.6 | no/β_2_-agonist | Electrochemical CO monitor | 4.8±0.5 |
|  | 17 | 7/10 | 47±5.15 | Moderate persistent | ---- | 71±7.5 | β_2_-agonist & ICS(L.M.H) |  | 2.5±0.4 |
|  | 15 | 6/9 | 38.9±4.2 | Severe persistent | ---- | 49±5.8 | oral steroids & ICS(H) |  | 4.3±0.4 |
|  | 10 | 6/4 | 34.1±2.8 | Healthy | ---- | ---- | ----- |  | 2.9±0.3 |
| Paredi  1999^#^  UK  [21] | 18 | - | 27±2 | Steroid-free | ---- | >80 | Not require ICS in the 3 months before the study | EC_50_-MICRO Smokerlyzer | 4.4±0.3 |
|  | 37 | 20/17 | 33±2.8 | Healthy | ---- | ---- | ---- |  | 2.1±0.2 |
| Uasuf  1999^#^  UK  [22] | 13 | 9/4 | 11.6±1.1 | Infrequent episodic | | 92.7±6.7 | no/β_2_-agonist | EC_50_ MICRO Smokerlyzer Breath hold for 20s | 1.39±0.18 |
|  | 16 | 11/5 | 11.3±0.5 | Persistent | | 87.3±3.9 | ICS(L.M.H) |  | 2.17±0.20 |
|  | 40 | 21/19 | 8.1±0.35 | Healthy | | ---- | ---- |  | 1.01±0.12 |
| Ece  2000^*^  Turkey  [23] | 30 | 35/19 | 4.5±1.7 | Steroid-treated | Mild-moderate persistent, Symptom free at least for 2 weeks | ---- | ICS(L) | Micro CO Meter | 0.96±0.95 |
|  | 24 |  |  | Steroid-free |  | ---- | β_2_-agonist |  | 1.78±1.53 |
|  | 235 | 137/98 | 4.4±2.3 | Healthy | ---- | ---- | ----- |  | 0.86±1.35 |
| Khatri  2001^#^  USA  [24] | 8 | 2/6 | 42±4 | Steroid-free | ---- | 71±5 | Not take oral antihistamine and inhaled anti-inflammation agents for more than 8 weeks | Siemens Ultramat 6 infrared analyzer Breath hold for 20s | 1.9±o.4 |
|  | 6 | 5/1 | 36±5 | Healthy | ---- | ---- | ---- |  | 1.8±0.2 |
| Yamaya  2001*  Japan  [25] | 20 | 11/9 | 63±18 | Mild | ---- | 87±9 | β_2_-agonist | EC_50_ Smokerlyzer Breath hold for 20s | 1.55±1.00 |
|  | 20 | 11/9 | 63±22 | Moderate | ---- | 85±9 | β_2_-agonist & ICS(L.M.H) |  | 1.55±1.10 |
|  | 15 | 8/7 | 64±19 | Stable severe | ---- | 81±8 | ICS(H) & oral steroids & β_2_-agonist |  | 4.53±5.52 |
|  | 16 | 8/8 | 65±16 | Unstable severe | ---- | 66±8 |  |  |  |
|  | 20 | 11/9 | 64±22 | Healthy | ---- | ---- | ---- |  | 1.15±0.8 |
| Zanconate  2002^#^  Italy  [26] | 19 | 19/11 | 10.5±0.5 | Persistent | With acute exacerbation | ---- | ICS(L) at least for 1 month | Crowcon TX Breath hold for 15s | 3.5±0.3 |
|  | 11 |  |  | Intermittent |  | ---- | no long term treatment |  | 2.6±0.3 |
|  | 21 | ---- | ---- | Healthy | ---- | ---- | ---- |  | 2.0±0.2 |
| Yilmaz  2003^*^  Turkey  [27] | 24 | 11/13 | 7.4±1.33 | Viral associated wheeze | Clinically stable for at least 4 weeks | 92.9±8.8 | β_2_-agonist on demand | piCO Smokerlyzer Breath hold for 20s | 1.19±0.4 |
|  | 36 | 25/11 | 9.2±2.0 | Persistent(Mild-Moderate) | With mild symptoms but not exacerbation | 80.4±12.0 | ICS(L.M.H) only or ICS(L.M.H) & montelukast |  | 2.05±0.9 |
|  | 23 | 38/19 | 10.8±2.6 | Seasonal asthma | Symptom free for at least 3 months | 93.1±10.8 | no/ICS(L.M.H)/ICS(L.M.H) & nasal corticosteroids |  | 1.45±0.6 |
|  | 34 |  | 9.4±2.2 |  | Mild symptoms for at least 4 weeks, no exacerbation | 84.3±16.9 |  |  | 2.30±0.80 |
|  | 45 | 25/20 | 10.4±2.8 | Healthy | ---- | ---- | ---- |  | 0.94±0.5 |
| Beck-Ripp  2004^#^  Ger  [28] | 15 | 6/9 | 11.6±0.7 | Steroid-free | ---- | 92.9±5.3 | ---- | LR2000 (electrochemical) | 4.99±0.45 |
|  | 20 | 11/9 | 11.5±0.7 | Healthy | ---- | ---- | ---- |  | 3.72±0.23 |
| Rosias  2004^#^  NED  [29] | 23 | ---- | 10.6±0.8 | Mild-moderate persistent | ACQ score 9.7±8.2 | 94.6±16.2 | ICS(L) for at least 6 months | EC_50_-MICO Smokerlyzer | 2.3±0.3 |
|  | 9 | ---- | 9.4±1.0 | Healthy | ---- | ---- | ---- |  | 2.1±0.7 |
| Antczak  2005^#^  UK  [17] | 8 | 5/3 | 34±2 | Mild | Stable in symptoms for at least 1 month | 70.5 | Stable with no changes in medication for at least 1 month,β_2_-agonist on demand | LR2000 (chemiluminescence) | 3.1±0.72 |
|  | 15 | 7/8 | 35±4 | Healthy | ---- | ---- | ---- |  | 3.3±2.06 |
| Ohara  2006^#^  Japan  [30] | 22 | 13/9 | 10±3 | Mild episodic | With acute exacerbation | ---- | no regular treatment | EC_50_-MICRO Smokerlyzer Breath hold for 15s | 5.1±0.4 |
|  | 29 | 19/10 | 10±3 | Infrequent episodic | Without acute exacerbation | ---- | no regular treatment |  | 1.1±0.1 |
|  | 188 | 113/75 | 10±1 | Healthy | ---- | ---- | ---- |  | 1.0±0.1 |
| Grover  2008^*^  India  [31] | 22 | ---- | >10 years  old | Miid persistent | Clinically stable | 94.32±7.85 | Not received ICS or oral steroids for at least 4 weeks | Mini Smokerlyzer Breath hold for 20s | 6.11±2.24 |
|  | 20 | ---- |  | Moderate persistent |  | 74.00±4.93 |  |  | 5.38±2.24 |
|  | 21 | ---- |  | Healthy | ---- | ---- | ---- |  | 3.86±1.35 |
