# Supplementary material for: Exhaled carbon monoxide in asthmatics: a meta-analysis
Source: Respir Res. 2010 Apr 30;11(1):50. doi: 10.1186/1465-9921-11-50 (PMC2874770; doi:10.1186/1465-9921-11-50)
Supplement: Additional file 3 — Studies included in the meta-analysis examining eCO levels in asthmatics before and after steroid treatment. Data are expressed as #mean ± SEM; *mean ± SD; +95%CI; M: male, F: female, n: the number of participants, ppm: parts per million, FEV1: forced expiratory volume in one second; eCO: exhaled carbon monoxide; ICS: inhaled corticosteroids; L: low dose of inhaled corticosteroids; M: medium dose of inhaled corticosteroids; H: high dose of inhaled corticosteroids. [file 1465-9921-11-50-S3.DOC]

| **study** | **n** | **M/F** | **Age** | **Clinical Feature** | **Measurement** | **Treatment** | **eCO before steroid treatment** | **eCO after steroid treatment** |
| --- | --- | --- | --- | --- | --- | --- | --- | --- |
| Zayasu,1997,[18] | 12 | 5/7 | 42±4 | Symptomatic, steroid-free, severe enough to require prophylactic treatment | EC_50_ Smokerlyzer, hold breath for 20s | ICS(L) for 4 weeks | 8.4±0.6 | 1.8±0.3 |
| Yamaya,1999#,Japan,[34] | 20 | 11/9 | 49±4 | Moderate, with acute exacerbation regular using ICS(L) | EC_50_ analysis, hold breath for 20s | Oral glucocorticoid treatment | 4.6±0.4 | 1.5±0.2 |
| Lim,2000 #, UK,[32] | 8 | ---- | ---- | Steroid-free, mild | EC_50_-MICRO Smokerlyzer hold breath for 20s | ICS(H) for 4 weeks | 3.28±0.39 | 3.17±0.36 |
| Yamaya,2001*,Japan,[25] | 15 | 8/7 | 64±19 | Stable severe | EC_50_ Smokerlyzer Breath hold for 20s | ICS(H) and oral prednisolone for 4 weeks | 7.6±8.9 | 4.3±5.7 |
|  | 16 | 8/8 | 65±16 | Unstable severe |  |  |  |  |
| Kharitonov,2002+,UK,[33] | 11 | ---- | 28 | Mild and occasional symptoms, atopic | LR2000,electrochemically | ICS(L,100ug/d) for 3 weeks | 3.4(2.8-4.0) | 2.8(1.9-3.7) |
|  | 11 | ---- |  |  |  | ICS(L,400ug/d) for 3 weeks | 3.2(2.6-3.9) | 2.4(1.6-3.3) |
| Zanconato,2002#,Italy,[26] | 30 | 19/11 | 10.5±0.5 | With acute exacerbation | Crowcon TX Breath hold for 15s | Oral prednisone for 5 days | 3.2±0.2 | 2.7±0.2 |
| Grover 2008^*^ India,[31] | 22 | ---- | >10 years old | Mild persistent, stable | Mini Smokerlyzer Breath hold for 20s | ICS(L) for 4 weeks | 6.11±2.24 | 5.36±2.06 |
|  | 20 | ---- |  | Moderate persistent, stable |  | ICS(M) for 4 weeks | 5.38±2.24 | 5.05±2.47 |
